# Neuronal Redox-Imbalance in Rett Syndrome Affects Mitochondria as Well as Cytosol, and Is Accompanied by Intensified Mitochondrial O_*2*_ Consumption and ROS Release

**DOI:** 10.3389/fphys.2019.00479

**Published:** 2019-04-30

**Authors:** Karolina Can, Christiane Menzfeld, Lena Rinne, Peter Rehling, Sebastian Kügler, Gocha Golubiani, Jan Dudek, Michael Müller

**Affiliations:** ^1^Center for Nanoscale Microscopy and Molecular Physiology of the Brain, University Medical Center Göttingen, Georg-August-University Göttingen, Göttingen, Germany; ^2^Zentrum Physiologie und Pathophysiologie, Institut für Neuro- und Sinnesphysiologie, Universitätsmedizin Göttingen, Georg-August-Universität Göttingen, Göttingen, Germany; ^3^Zentrum Biochemie und Molekulare Zellbiologie, Institut für Zellbiochemie, Universitätsmedizin Göttingen, Georg-August-Universität Göttingen, Göttingen, Germany; ^4^Klinik für Neurologie, Universitätsmedizin Göttingen, Georg-August-Universität Göttingen, Göttingen, Germany; ^5^Institute of Chemical Biology, Ilia State University, Tbilisi, Georgia

**Keywords:** oxidative stress, reactive oxygen species, disease progression, methyl-CpG binding protein 2 encoding gene (mouse), hippocampus, cortex, heart, reduction-oxidation sensitive green fluorescent protein 1

## Abstract

Rett syndrome (RTT), an X chromosome-linked neurodevelopmental disorder affecting almost exclusively females, is associated with various mitochondrial alterations. Mitochondria are swollen, show altered respiratory rates, and their inner membrane is leaking protons. To advance the understanding of these disturbances and clarify their link to redox impairment and oxidative stress, we assessed mitochondrial respiration in defined brain regions and cardiac tissue of male wildtype (WT) and MeCP2-deficient (*Mecp2^-/y^*) mice. Also, we quantified for the first time neuronal redox-balance with subcellular resolution in cytosol and mitochondrial matrix. Quantitative roGFP1 redox imaging revealed more oxidized conditions in the cytosol of *Mecp2^-/y^* hippocampal neurons than in WT neurons. Furthermore, cytosol and mitochondria of *Mecp2^-/y^* neurons showed exaggerated redox-responses to hypoxia and cell-endogenous reactive oxygen species (ROS) formation. Biochemical analyzes exclude disease-related increases in mitochondrial mass in *Mecp2^-/y^* hippocampus and cortex. Protein levels of complex I core constituents were slightly lower in *Mecp2^-/y^* hippocampus and cortex than in WT; those of complex V were lower in *Mecp2^-/y^* cortex. Respiratory supercomplex-formation did not differ among genotypes. Yet, supplied with the complex II substrate succinate, mitochondria of *Mecp2^-/y^* cortex and hippocampus consumed more O_2_ than WT. Furthermore, mitochondria from *Mecp2^-/y^* hippocampus and cortex mediated an enhanced oxidative burden. In conclusion, we further advanced the molecular understanding of mitochondrial dysfunction in RTT. Intensified mitochondrial O_2_ consumption, increased mitochondrial ROS generation and disturbed redox balance in mitochondria and cytosol may represent a causal chain, which provokes dysregulated proteins, oxidative tissue damage, and contributes to neuronal network dysfunction in RTT.

## Introduction

Rett syndrome (RTT) is a progressive neurodevelopmental disorder. It primarily affects females, who show the first obvious symptoms within 6–18 months after birth. Among the characteristics are a regression of mental and physical development as well as severe disabilities such as breathing disturbances, loss of speech, autistic features, cognitive impairment, and epilepsy (Rett, 1966; Hagberg et al., 1983; Chahrour and Zoghbi, 2007). In the majority of cases, RTT is caused by spontaneous mutations in the X-chromosomal *MECP2* gene encoding for the transcriptional regulator MeCP2 (Amir et al., 1999).

Patient and rodent studies confirm that mitochondria are morphologically and functionally affected in RTT (Eeg-Olofsson et al., 1990; Belichenko et al., 2009; Großer et al., 2012; Gold et al., 2014; Park et al., 2014; Valenti et al., 2014; Shulyakova et al., 2017). Human skeletal muscle and frontal lobe biopsy samples revealed swollen mitochondria with vacuolizations and granular inclusions (Ruch et al., 1989; Eeg-Olofsson et al., 1990; Cornford et al., 1994). In male Rett mice carrying a knockout mutation of the *Mecp2* gene (*Mecp2^-/y^* mice), we previously observed that hippocampal mitochondria show less negative membrane potentials and increased FAD/NADH baseline-ratios, which indicates an intensified degree of oxidation and increased levels of ROS (Großer et al., 2012; Müller and Can, 2014). Also, brain ATP content is affected. Higher resting ATP levels with increased ATP turnover rates were reported for neonatal hippocampal *Mecp2^-/y^* neurons (Toloe et al., 2014), whereas whole brain studies on adult symptomatic male and female Rett mice detected reduced ATP concentrations (Saywell et al., 2006; De Filippis et al., 2015; Valenti et al., 2017).

In view of these findings, also specific changes in mitochondrial respiration are to be expected. The mitochondrial respiratory chain consists of four complexes, CI, CII, CIII, and CIV, which are transporting electrons from reducing equivalents (NADH+H^+^, FADH_2_) to molecular oxygen. This electron transport extrudes protons across the inner membrane, and the resulting membrane potential then drives ATP synthesis by the F_1_F_o_-ATP synthase (CV) (Mitchell, 1961). Indeed, Rett patients show lower expression levels of cytochrome *c* oxidase subunit I as well as reduced enzymatic activities of cytochrome *c* oxidase and succinate cytochrome *c* reductase (Coker and Melnyk, 1991; Gibson et al., 2010). Also in Rett mice, lowered enzymatic activities of the respiratory complexes are evident in brain and skeletal muscle (Kriaucionis et al., 2006; Gold et al., 2014; De Filippis et al., 2015; Valenti et al., 2017). In concert with altered O_2_ consumption rates (Kriaucionis et al., 2006) these changes may easily culminate in reduced brain ATP contents (Saywell et al., 2006; De Filippis et al., 2015; Valenti et al., 2017).

Nevertheless, detailed information on the very brain regions affected and on the exact time course of mitochondrial changes in RTT is sparse. Another issue still to be addressed, is whether besides differential protein levels and activities also altered protein/protein interactions may contribute to the mitochondrial dysfunction in RTT. The mitochondrial respiratory chain forms large supercomplexes in which CI binds to a dimer of CIII and several copies of CIV (Schägger and Pfeiffer, 2000; Lenaz and Genova, 2012; Wu et al., 2016). This supercomplex formation guarantees an efficient energy transduction within the respiratory chain, prevents energy leakage, and dampens ROS production. Pathological changes in supercomplex structure were reported for many disease models (Wallace, 1999; DiMauro and Schon, 2003; Pacheu-Grau et al., 2015; Dudek et al., 2016) – but at present it is unclear whether this may be the case also in RTT.

Another aspect is that mitochondria are a prominent source of ROS. During electron transport from CI to CIV approximately 2–5% of electrons escape and diffuse directly to O_2_, thereby generating superoxide (Boveris and Chance, 1973; Votyakova and Reynolds, 2001; Brand, 2010). Hence, the increased mitochondrial activity and ATP turnover in Rett mice (Kriaucionis et al., 2006; Großer et al., 2012; Toloe et al., 2014) should be associated with increased ROS generation. Indeed, an intensified H_2_O_2_ production was observed in isolated mitochondria of female Rett mouse brains (De Filippis et al., 2015; Valenti et al., 2017). It appears to contribute to the systemic oxidative burden and the oxidative tissue damage in RTT (De Felice et al., 2009; De Felice et al., 2014), which is assumed to drive the disease progression in RTT (De Felice et al., 2012; Großer et al., 2012; Müller and Can, 2014; Müller, 2019). Yet again, these studies were performed on full brain and do not provide any brain region-specific or even subcellular insights. Furthermore, it still has to be clarified in detail, how the mitochondrial alterations are mechanistically linked to redox-imbalance, oxidative stress, and neuronal network dysfunction in RTT.

Therefore, we took a closer look at redox balance specifically in neurons, and we quantified redox conditions in their cytosolic and mitochondrial compartment, by using the advanced, genetically encoded redox sensor roGFP1. Furthermore, we assessed multiple aspects of mitochondrial physiology in defined brain regions of Rett mice to unravel the interplay of mitochondrial alterations and cellular redox balance in this neurodevelopmental disorder. In particular, we focused on hippocampal and cortical tissue, as these brain areas are metabolically highly demanding, very vulnerable to oxidative stress, and essentially underlie complex cognitive functions. By means of quantitative redox imaging and comprehensive biochemical protein analyzes, we confirm that the redox impairment in RTT affects neuronal cytosol as well as mitochondrial matrix. Only moderate decreases in the expression of respiratory complex constituents were evident in mitochondria of *Mecp2^-/y^* hippocampus and cortex. Nevertheless, their O_2_ consumption rate was increased in the presence of CII substrates. Also, an intensified ROS generation was confirmed for mitochondria of *Mecp2^-/y^* hippocampus and cortex.

## Materials and Methods

As a mouse model for RTT, we chose *Mecp2* knockout mice [B6.129P2(C)-*Mecp2^tm1.1Bird^*] (Guy et al., 2001). In order to ensure uniform conditions, i.e., a total lack of MeCP2 in each single cell studied by our cell-based and tissue analyzes, only male Rett mice (*Mecp2^-/y^*) were used. They develop symptoms earlier, present a more severe phenotype, and were compared with their male WT siblings. Optimized breeding of Rett mice could be achieved with WT (NMRI line) foster mice only, which took better care of the offspring than the female Rett mice (*Mecp2^+/-^*). All experiments were in accordance with German regulations and authorized by the Office of Animal Welfare of the University Medical Center Göttingen as well as by the Lower Saxony State Office for Consumer Protection and Food Safety. Mouse genotypes were disclosed only after data collection and analysis. Yet, for *Mecp2^-/y^* mice, the genotype eventually became obvious due to their characteristic phenotype and behavior.

### Solutions

Unless stated differently, all chemicals were obtained from Sigma-Aldrich. Growth medium used for organotypic slice cultures consisted of minimum essential medium (MEM, Invitrogen) supplemented with 5% FCS, 0.5 mM L-glutamine, 20 μl/ml B27 50× supplement (Invitrogen), 2 μM cytosine arabinoside, and 100 μg/ml penicillin-streptomycin (Biochrom). ACSF contained 130 mM NaCl, 3.5 mM KCl, 1.25 mM NaH_2_PO_4_, 24 mM NaHCO_3_, 1.2 mM CaCl_2_, 1.2 mM MgSO_4_ and 10 mM dextrose, and was aerated constantly with 95% O_2_ plus 5% CO_2_ (carbogen) to ensure a stable pH of 7.4 as well as a proper O_2_ supply of the tissue under investigation. The redox stimulants H_2_O_2_ (30% aqueous stock solution), DEDTC, and 1,4-dithio-DL threitol (DTT, Fluka) were added to the ACSF immediately before use. AMC was first dissolved as 20 mM stock solution in absolute ethanol and stored at -20°C. The oxidation-sensitive dyes H_2_DCFDA (2′,7′-dichlorodihydrofluorescein diacetate, Invitrogen) and Amplex UltraRed (Invitrogen) were dissolved in dimethyl sulfoxide as 100 mM and 10 mM stock solutions, respectively, and stored at -20°C.

### Hippocampal Slice Cultures

Organotypic slice cultures were prepared from neonatal male mice at PD 3–5, as described earlier (Stoppini et al., 1991; Großer et al., 2012). After decapitation, the brain was removed and placed in ice-cold Hanks’ balanced salt solution. Isolated hippocampi were cut into 350 μm-thick slices (McIlwain tissue chopper, Stoelting Co.), placed on the membranes of six-well culture plates (Transwell Permeable Support, Corning), and incubated under interface conditions at 37°C in a 5% CO_2_-atmosphere. Each well contained 1 ml growth medium, half of which was replaced every 2–3 days.

### Optical Recordings and Transduction Procedures

Cellular redox conditions were monitored with a CCD-camera imaging system. It was controlled by TILLvisION 4.0.1 software (TILL Photonics) and consisted of a xenon light source (Polychrome II; Till Photonics), a sensitive CCD camera (Imago QE; PCO Imaging), and an upright fluorescence microscope (Axioskop I; Zeiss) with a 63× 1.0 NA water immersion objective (Plan-Apochromat; Zeiss). For the imaging experiments, slice cultures were placed with their support membranes in a submersion-style chamber and superfused with pre-warmed ACSF (37°C, 4.5 ml/min).

Neuronal redox balance was quantified with the excitation-ratiometric optical redox sensor roGFP1 (Dooley et al., 2004; Hanson et al., 2004). This optical probe does not detect a particular reactive oxygen or nitrogen species, but rather reports thiol redox balance, and it responds reversibly to redox modulation. Hence, roGFP1 senses – similar to numerous other cellular proteins (Weerapana et al., 2010) – the subcellular thiol redox conditions. In detail, oxidation increases light absorption at 390 nm and decreases absorption at 470 nm; reduction causes opposite responses. Therefore, we excited roGFP1 alternatingly at 395 and 470 nm, and calculated the resulting fluorescence ratio R (Funke et al., 2011; Großer et al., 2012); as optical filters we chose a 460–480 nm bandpass excitation filter, a 495 nm dichroic mirror, and a 525–550 nm bandpass emission filter. Among the advantages of this excitation-ratiometric approach are highly stable recordings as well as improved response dynamics, and the ratiometric redox responses obtained are independent of the expression level of the redox sensor. Nevertheless, quantitative analyses demand that those roGFP1 ratios are determined which correspond to full oxidation (R_ox_) and full reduction (R_red_), as induced by saturating doses of H_2_O_2_ (5 mM), and DTT (10 mM), respectively (Hanson et al., 2004; Funke et al., 2011; Wagener et al., 2016). Once calibrated, the relative degree of oxidation OxD_roGFP1_ can be determined for the respective experimental conditions [see (Meyer and Dick, 2010)]:

$${\text{OxD}}_{\text{roGFP}1}=\frac{\text{R}-{\text{R}}_{\text{red}}}{\frac{\text{F}470_{\text{ox}}}{\text{F}470_{\text{red}}}\;\left(R_{ox}-R\right)\;+\;\left(R-R_{red}\right)}$$

Based on OxD_roGFP1_ and the standard redox potential of roGFP1 (E^0^′ = -291 mV), the Nernst equation then yields the corresponding roGFP1 redox potentials (Hanson et al., 2004; Meyer and Dick, 2010; Wagener et al., 2016):

$${\text{E}}_{\text{roGFP}1}={\text{E}}^{0}\text{′roGFP}1-\frac{\text{RT}}{2\text{F}}\text{ln}\left(\frac{1-{\text{OxD}}_{\text{roGFP}1}}{{\text{OxD}}_{\text{roGFP}1}}\right)$$

After confirming reliable responses of roGFP1 in hippocampal cells and slices (Funke et al., 2011; Großer et al., 2012), we developed viral constructs (AAV6) and placed roGFP1 under the human synapsin 1 promoter, to optimize the transduction efficiency and to ensure a neuron-specific expression. Without further targeting sequences, roGFP1 is expressed in the cytosol (cyto-roGFP1). Its reliable expression in mitochondrial matrix (mito-roGFP1) is guaranteed by the included mitochondrial targeting sequence of subunit VIII of cytochrome *c* oxidase (Can et al., 2017). Slice cultures were transduced on DIV 3–4, by applying 2.5 μl of the vector (1:50 dilution in PBS; undiluted titer 1.3 × 10^8^ particles/μl) onto each slice. Redox imaging experiments were then performed between DIV 9–15.

### Tissue Preparation From Adult Mice

Adult male mice (PD43-49) were ether anesthetized, decapitated, and the brain removed as described earlier (Fischer et al., 2009). The heart was isolated as well, and the collected tissue was processed further to prepare mitochondrial suspensions. Excess tissue was cryopreserved in liquid N_2_ and stored at -80°C for later use.

### Mitochondrial Suspensions

Mitochondrial isolation was performed as described before, with some modifications (Argan et al., 1983). The rigid cardiac tissue was first sliced into small pieces, transferred into 1 ml isolation buffer and minced in a blender (Ultra Thurax T8, IKA Labortechnik). Hippocampal and cortical tissue was transferred into isolation buffer (20 mM HEPES pH 7.6, 220 mM mannitol, 70 mM sucrose, 1 mM 2,2′,2′′,2′′′-(1,2-ethanediyldinitrilo)-tetraacetic acid (EDTA), 0.5 mM PMSF, and homogenized (Potter S, Sartorius) at 1 × 300, 1 × 500, and 25 × 700 rpm. After removing cell debris by centrifugation (800 g, 15 min, 4°C) supernatants were pooled and centrifuged again for 30 min to remove remaining debris. Part of the supernatant was cryopreserved as whole-cell lysate for later protein analyzes. Remaining supernatant was centrifuged at 10,000 g (10 min, 4°C) to spin down the mitochondrial fraction, and the pellets were resuspended in isolation buffer. Final volume and protein amounts were determined by Bradford assay.

### Protein Analysis

Proteins of whole cellular lysates were separated by SDS-PAGE (12.5% SDS-PAGE), loading 10–20 μg/lane protein. Blue native (BN) gel electrophoresis was performed as reported earlier (Wittig et al., 2006; Vukotic et al., 2012). For Western blot analysis 30 μg/lane of isolated mitochondria were used. In-gel activity staining required higher protein amounts (CI: 50 μg/lane, CIV: 100 μg/lane, and CV: 150 μg/lane). Mitochondrial membranes were solubilized in lysis buffer [20 mM Tris/HCl pH7.4, 0.1 mM EDTA, 50 mM NaCl, 10% (v/v) glycerol, 1% (w/v) digitonin, 1 mM PMSF] and incubated for 30 min at 4°C while shaking constantly. Non-solubilized material was removed by centrifugation (Eppendorf 5415R, 16,000 g, 5 min, 4°C). Supernatants were transferred into a new sample tube and mixed with 10× loading buffer [5% (w/v) Coomassie Brilliant Blue G-250, 500 mM 𝜀-amino-n-caproic acid diluted in 100 mM Bis-Tris pH7.0]. Samples were separated on a 4–14% poly-acrylamide gradient gel, using a cathode buffer (50 mM Tricine pH7.0, 15 mM Bis-Tris, 0.02% Coomassie G-250) and an anode buffer (50 mM Bis-Tris/HCl pH7.0) (Dekker et al., 1997).

For Western blots, standard methods were used to transfer proteins to polyvinylidene fluoride membranes. After blocking (5% skimmed milk diluted in TBS containing 0.1% Tween), membranes were probed with primary antibodies. Anti-NADH dehydrogenase (ubiquinone) 1 beta subcomplex 8 (NDUFB8), anti-succinate dehydrogenase A (SDHA, Cell Signaling Technology), anti-ubiquinol-cytochrome *c* reductase, Rieske iron-sulfur polypeptide 1 (Rieske), anti-cytochrome *c* oxidase subunit I (COX1), ATP synthase subunit beta (AtpB, Antikoerper-online), and anti-VDAC3 were produced in rabbit. Anti-glyceraldehyde-3-phosphate dehydrogenase (GAPDH, 5G4-6C5, Biotrend) was produced in mice. Secondary antibodies were coupled to horseradish peroxidase (Dianova). Chemiluminescence (ECL^TM^ Western blot detection reagent, GE or Clarity^TM^ Western ECL substrate, BioRad) was detected using films (Medix XBU Medical X-Ray Film, Foma Bohemia) or imaged with a camera (Image Reader LAS-1000, Fujifilm). Signal intensities were analyzed by Aida Image Analyzer V.3.10.

In-gel catalytic activity of native respiratory chain complexes separated by BN-PAGE was rated as reported earlier (Zerbetto et al., 1997; Wittig et al., 2007). CI activity was visualized with 2.5 mg/ml NTB in staining solution (0.1 mg/ml NADH and 5 mM Tris, pH 7.4) at 37°C until staining became visible. For CIV staining, cytochrome *c*, reduced with 15 mg/ml Na-dithionite, was added at a concentration of 1 mg/ml to the staining solution containing 0.5 mg/ml diaminobenzidine in 50 mM KPi, pH 7.4. CV staining was performed in 35 mM Tris–HCl pH 8.3, 270 mM glycine, 14 mM MgSO_4_ 0.2% Pb(NO_3_)_2_, and 8 mM ATP. Band intensities were quantified by ImageQuant TL 1D Version 7.0 (GE Health Care).

### Mitochondrial O_2_ Consumption

For quantitative analysis of O_2_ consumption, a Seahorse XF96 Extracellular Flux Analyzer (Seahorse Bioscience) was used. Isolated mitochondria were resuspended in assay buffer (MAS, 70 mM sucrose, 220 mM mannitol, 2 mM HEPES, 10 mM KH_2_PO_4_, 5 mM MgCl_2_, 1 mM EGTA, and 0.2% BSA, pH 7.4), and loaded to a 96-well sample plate. A centrifugation step (2,000 g, 20 min, 4°C, Allegra X-15R, Beckman Coulter) ensured that mitochondria adhered to the bottom of the wells. Basal respiration was measured at 37°C, in 180 μl MAS containing 2 mM malate + 10 mM pyruvate or 4 mM succinate as substrate. Subsequent consecutive measurements were performed after reoxygenation and automated administration of 12 mM ADP, 1.5 μM oligomycin, 4 μM FCCP, and 4 μM antimycin A + 2 μM rotenone. Data were analyzed with the XF96 Software (Version 1.3.0.79).

### ROS Formation by Isolated Mitochondria

Mitochondrial ROS formation was probed with the oxidation-sensitive dyes H_2_DCFDA (100 μM) and Amplex UltraRed (10 μM). These cuvette assays are standard approaches, which define ROS formation in response to mitochondrial challenges and/or in transgenic mouse models (Mattiasson, 2004; Rönnbäck et al., 2016). In the case of AmplexRed, H_2_O_2_ is detected in a coupled reaction with horseradish peroxidase (1 U/ml) (Zhou et al., 1997; Komary et al., 2010). Isolated mitochondria (40 μg) were resuspended in MAS buffer for native conditions or in a denaturing buffer (20 mM Tris, 150 mM NaCl, 0.1% Triton, pH 7.4). The oxidation of H_2_DCFDA and Amplex UltraRed is irreversible. Accordingly, accumulation of the oxidized dye results in a continuous increase in fluorescence emission. For H_2_DCFDA it was monitored with a fluorescence spectrophotometer (Hitachi F-7000) at 495 nm excitation and 525 nm emission wavelengths, and for Amplex UltraRed a multimode spectrophotometer (flx-Xenius XM, SAFAS Monaco) with 555 nm excitation and 581 nm emission wavelengths was used.

### Body Temperature Measurements

The core body temperature of mice was determined with a rectal probe (MLT451) and a temperature controller (TC1000, ADInstruments) on three consecutive days. The first day served to adapt the mice to the procedure; the temperatures measured on the second and third day were averaged for each mouse.

### Statistics

Throughout the manuscript, data are presented as mean ± standard deviation. For each diagram it is indicated whether the number of experiments (*n*) equals the number of cells analyzed (optical imaging) or the number of mice studied (biochemical assays, tissue samples). Significance of the differences observed was determined by unpaired two tailed student’s *t*-test. Genotype-related differences are marked by asterisks (^∗∗∗^*p* < 0.001, ^∗∗^*p* < 0.01, ^∗^*p* < 0.05) and genotype-matched differences among cell compartments are identified by crosshatches (^###^*p* < 0.001, ^##^*p* < 0.01, ^#^*p* < 0.05). In the case of normalized data, a one-sample *t*-test served to compare the observed changes to control conditions (defined as unity, i.e., 1.0 or 100%) (Bailey, 1992).

## Results

### Redox Balance in Mitochondrial Matrix and Cytosol

**FIGURE 1:**
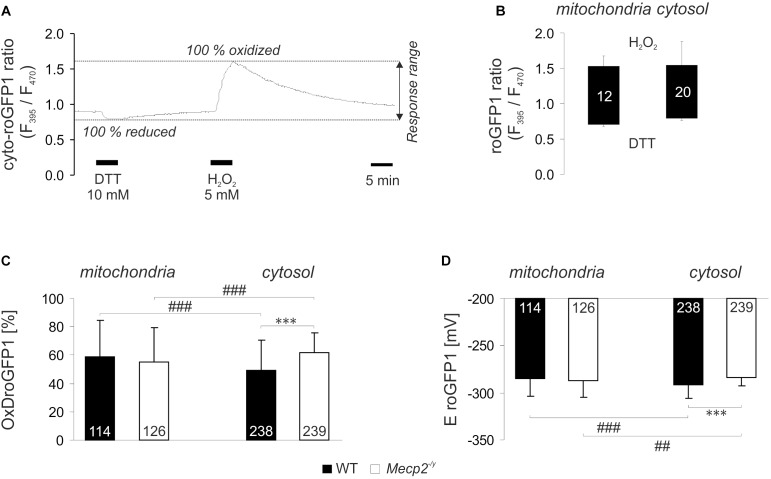
Reduction-oxidation sensitive green fluorescent protein 1 (roGFP1) response calibration reveals genotypic and compartmental redox differences at baseline resting conditions. **(A)** Dynamic response of cyto-roGFP1 to saturating oxidation and reduction, as recorded from a WT slice culture. **(B)** The box plot summarizes the response ranges of mito-roGFP1 and cyto-roGFP1 determined during the calibration experiments. The bottom of each box represents the fully reduced state evoked by 10 mM DTT, the ceiling indicates the fully oxidized level induced by 5 mM H_2_O_2_. Error bars in this and all other figures are standard deviations; the number of cells analyzed is reported for each bar. The similarity of the determined response ranges confirms that roGFP reliably functions in both, cytosol and mitochondrial matrix. **(C)** OxD_roGFP1_ confirms more oxidized baseline conditions in the cytosol of *Mecp2^-/y^* hippocampal neurons than in WT neurons. Mitochondrial redox baselines did not differ among genotypes. Statistically significant differences between WT and *Mecp2^-/y^* neurons are indicated by asterisks (^∗∗∗^*p* < 0.001). Genotype-matched differences among mitochondria and cytosol are indicated by crosshatches (^###^p < 0.001). Pairwise comparisons were run as unpaired two-tailed *t*-tests; the number of cells studied is included in each bar. **(D)** These genotypic and compartmental redox differences are also evident from the calculated roGFP1 reduction potentials (E_roGFP1_; ^##^*p* < 0.01).

Our previous analyses revealed a cytosolic redox-imbalance in *Mecp2^-/y^* hippocampus (Großer et al., 2012). To compare in detail the redox conditions in mitochondria and cytosol of *Mecp2^-/y^* neurons in organotypic hippocampal slice cultures, we analyzed the resting-baseline (steady-state) redox conditions in both cell compartments, and recorded also their dynamic responses to various acute redox-stimuli. For these tasks, we used our recently established viral constructs. They express roGFP1 under the control of the synapsin 1 promoter, mediating a neuron specific expression, and proper subcellular targeting to either cytosol (cyto-roGFP1) or mitochondrial matrix (mito-roGFP1) (Can et al., 2017). RoGFP1 enables quantitative analyses of subcellular redox conditions, but this requires detailed response calibrations for each cell-compartment. Therefore, with saturating doses of H_2_O_2_ (5 mM) and DTT (10 mM) we defined those ratiometric values which correspond to full roGFP1 oxidation and reduction, respectively (Figure 1A). H_2_O_2_ (7 min) increased the mito-roGFP1 ratio to 1.52 ± 0.16, and DTT decreased it to 0.68 ± 0.02 (*n* = 12, Figure 1B). As cyto-roGFP1 responded more promptly, the redox-stimulants were applied for 5 min only, but very similar calibration values were obtained as in mitochondria (*n* = 20; Figure 1B).

Based on these calibrations, the relative degrees of roGFP1 oxidation (OxD_roGFP1_) and E_roGFP1_ were calculated for steady-state resting baseline conditions. To define genotype-related differences, the respective cell compartments were compared among WT and *Mecp2^-/y^* slices. Furthermore, to identify subcellular, i.e., compartment-specific differences in a given genotype, cytosol and mitochondrial matrix were compared to each other. This revealed that in WT neurons, mitochondrial matrix was more oxidized than their cytosol. Yet, mitochondrial redox baselines did not differ among WT and *Mecp2^-/y^* slices. In contrast, cytosolic redox balance was more oxidized in *Mecp2^-/y^* than in WT neurons (Figure 1C). As a result, the cytosol of *Mecp2^-/y^* neurons was even more oxidized than their mitochondria. These compartmental and genotypic differences are also reflected by the E_roGFP1_ (Figure 1D).

**FIGURE 2:**
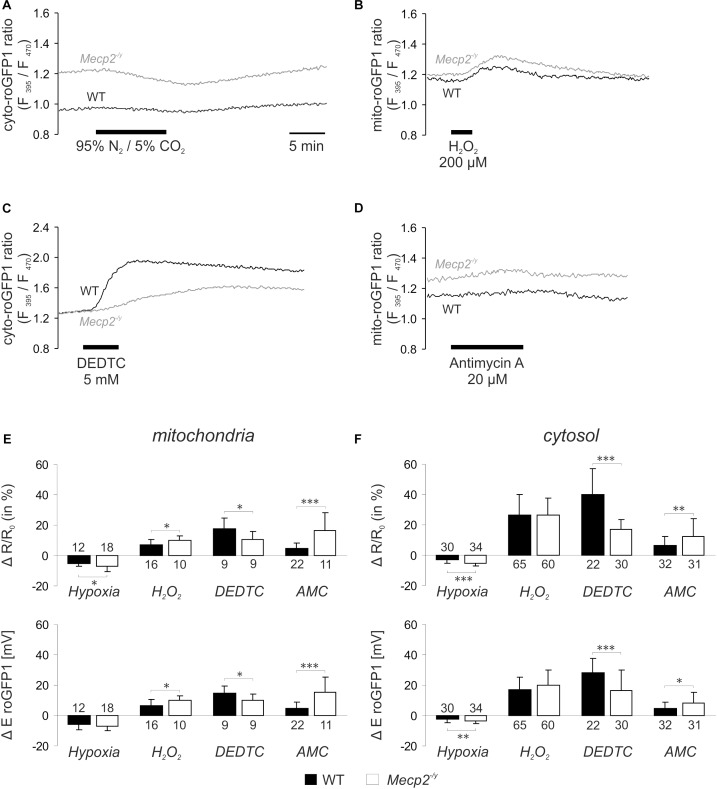
Acute redox challenge uncovers a more fragile redox homeostasis in mitochondrial matrix and cytosol of *Mecp2^-/y^* hippocampal neurons. **(A)** As revealed by the imaging of subcellular redox dynamics, O_2_ withdrawal evoked a reducing shift in the cytosolic roGFP1 fluorescence ratio, which was more intense in *Mecp2^-/y^* neurons. Time scaling also applies to the following traces. **(B)** Low-dose H_2_O_2_ induced a more pronounced oxidation in *Mecp2^-/y^* mitochondria. **(C)** The cytosol of *Mecp2^-/y^* neurons responded less intensely to DEDTC, a blocker of SOD1. **(D)** Block of CIII by AMC elicited a more pronounced oxidation in *Mecp2^-/y^* mitochondria. **(E)** Summary of the redox alterations in mitochondrial matrix. Plotted are changes in mito-roGFP1 ratio and in the mito-roGFP1 reduction potential. Asterisks indicate statistically significant differences as compared to WT in unpaired two-tailed *t*-tests (^∗∗∗^*p* < 0.001, ^∗∗^*p* < 0.01, ^∗^*p* < 0.05). The number of cells studied is reported for each bar. **(F)** Summarized redox responses of the cytosolic compartment (cyto-roGFP1).

Next, we assessed the dynamic redox responses of both cell compartments to defined challenges. As irregular respiration with intermittent hypoxia is a hallmark of RTT, the effect of O_2_ shortage was determined. O_2_ withdrawal (N_2_/CO_2_-aerated ACSF, 10 min), evoked a reducing shift in roGFP1 ratio, which was more intense in *Mecp2^-/y^* mitochondria and *Mecp2^-/y^* cytosol than in the WT compartments (Figure 2A,E,F). Oxidant challenge was induced by low-dose H_2_O_2_ or by inhibition of the scavenging enzyme SOD1 (Cu/Zn SOD). H_2_O_2_ (200 μM, 3–5 min) elicited an oxidizing shift in roGFP1 ratio, which was more pronounced in *Mecp2^-/y^* than in WT mitochondria, but did not differ among *Mecp2^-/y^*, and WT cytosol (Figure 2B,E,F). Blocking SOD1 by DEDTC (Iqbal and Whitney, 1991; Dumay et al., 2006) (5 mM, 5 min) induced an oxidation of roGFP1, which was consistently more pronounced in WT mitochondria and cytosol than in the respective *Mecp2^-/y^* compartments (Figure 2C,E,F). Mitochondrial impairment was mimicked by the CIII-blocker AMC (20 μM, 10 min), and it evoked a slow oxidizing shift, which was more intense in *Mecp2^-/y^* mitochondria and cytosol as compared to the WT compartments (Figure 2D–F).

### Mitochondrial Mass and Expression Levels of Respiratory Complexes

**FIGURE 3:**
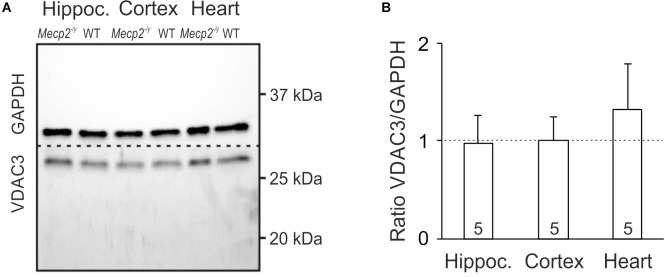
Mitochondrial mass is not altered in *Mecp2^-/y^* hippocampus and cortex. **(A)** In Western blot analyses, VDAC3 served as mitochondrial marker. Its expression was referred to GAPDH, and tissue lysates of hippocampus, cortex, and heart were compared among WT and *Mecp2^-/y^* siblings. Dashed line indicates the membrane cut after protein blotting. **(B)** In densitometric analyzes genotypic differences for hippocampus and cortex were not found. Mitochondrial mass tended to be higher though in *Mecp2^-/y^* heart (*n* = 5 mice each). Plotted VDAC3/GAPDH ratios are normalized to WT conditions (defined as unity, see dashed line), statistical significance was assessed by one-sample *t*-tests.

An increased cellular density of mitochondria may be a potential cause of the oxidative burden in RTT. Therefore, we asked whether mitochondrial mass differs in WT and *Mecp2^-/y^* mice. As mitochondrial marker we chose VDAC3, an integral membrane protein of the outer mitochondrial membrane; the cytosolic protein GAPDH served as loading control. Clear cellular lysates of hippocampus, cortex and heart (*n* = 5 each genotype) were separated by SDS-PAGE, and both proteins detected in the same sample by Western blot analysis (Figure 3A). Normalizing the expression of VDAC3 to GAPDH, did not reveal any differences in mitochondrial mass of adult *Mecp2^-/y^* hippocampus or cortex as compared to WT. Only a trend toward increased mitochondrial mass was evident in *Mecp2^-/y^* heart, which did, however, not reach the level of significance (*p* = 0.101; Figure 3B).

**FIGURE 4:**
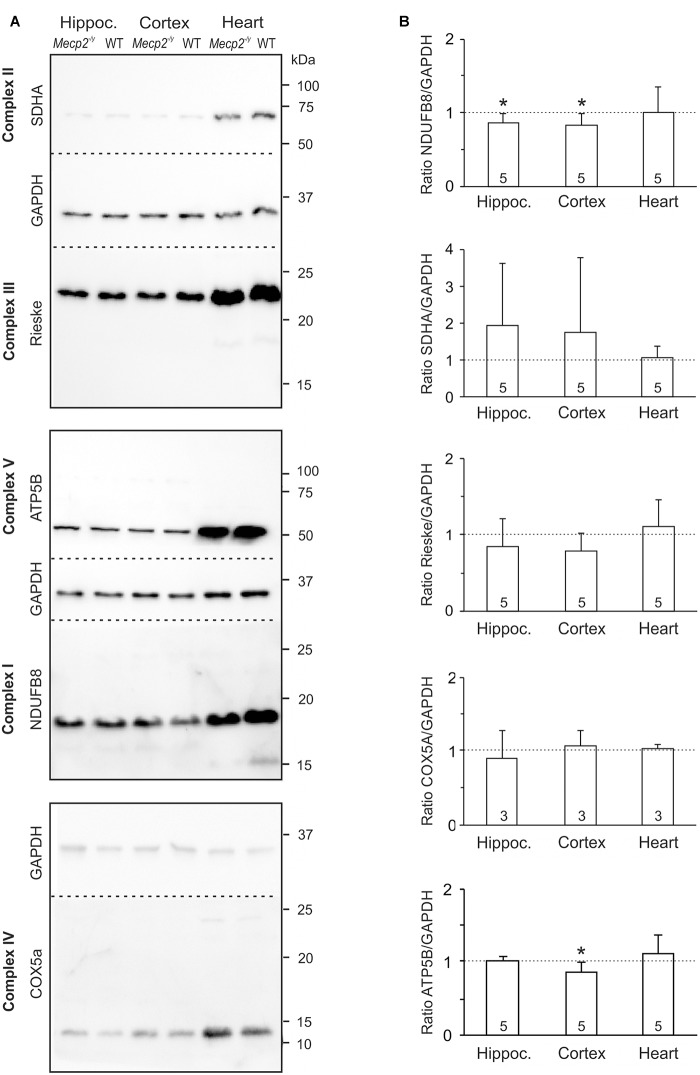
Protein expression levels of NDUFB8 and ATP5B differ only slightly between *Mecp2^-/y^* and WT mice. **(A)** Protein lysates were separated by SDS gel electrophoresis and analyzed by Western blotting, using antibodies specific against NDUFB8 (CI), SDHA (CII), Rieske (CIII), COX5A (CIV), ATP5B (CV), and GAPDH. Dashed lines indicate the membrane cut after protein blotting. The displayed blots of CII and CIII are from the same experiment and therefore share a common loading control; this also applies to CV and CI. **(B)** Expression levels of the proteins of interest were quantified by densitometry, normalized to GAPDH within the same tissue sample and then referred to the WT samples (defined as unity, see dashed line). The number of samples (mice) analyzed is reported; asterisks mark statistically significant differences as compared to WT in one sample *t*-tests (^∗^*p* < 0.05).

As a reduced enzymatic activity of key mitochondrial functions is evident in RTT (Coker and Melnyk, 1991; Kriaucionis et al., 2006; Gibson et al., 2010; Gold et al., 2014; De Filippis et al., 2015; Valenti et al., 2017), we analyzed steady state levels of constituents of the mitochondrial respiratory chain. Tissue lysates were separated by SDS-PAGE and protein levels of core constituents of the respiratory chain were analyzed by Western blotting (Figure 4A). All complexes were detectable in the WT and *Mecp2^-/y^* samples, but normalization to GAPDH content did not reveal any genotypic differences in the protein levels of CII, CIII, and CIV (Figure 4B). In *Mecp2^-/y^* hippocampus and cortex, however, CI protein levels (NDUFB8) were slightly lower than in WT. Furthermore, ATP-synthase (CV, ATP5B) was slightly less expressed in *Mecp2^-/y^* cortex (Figure 4B).

### Structure of Mitochondrial Respiratory Chain Complexes

**FIGURE 5:**
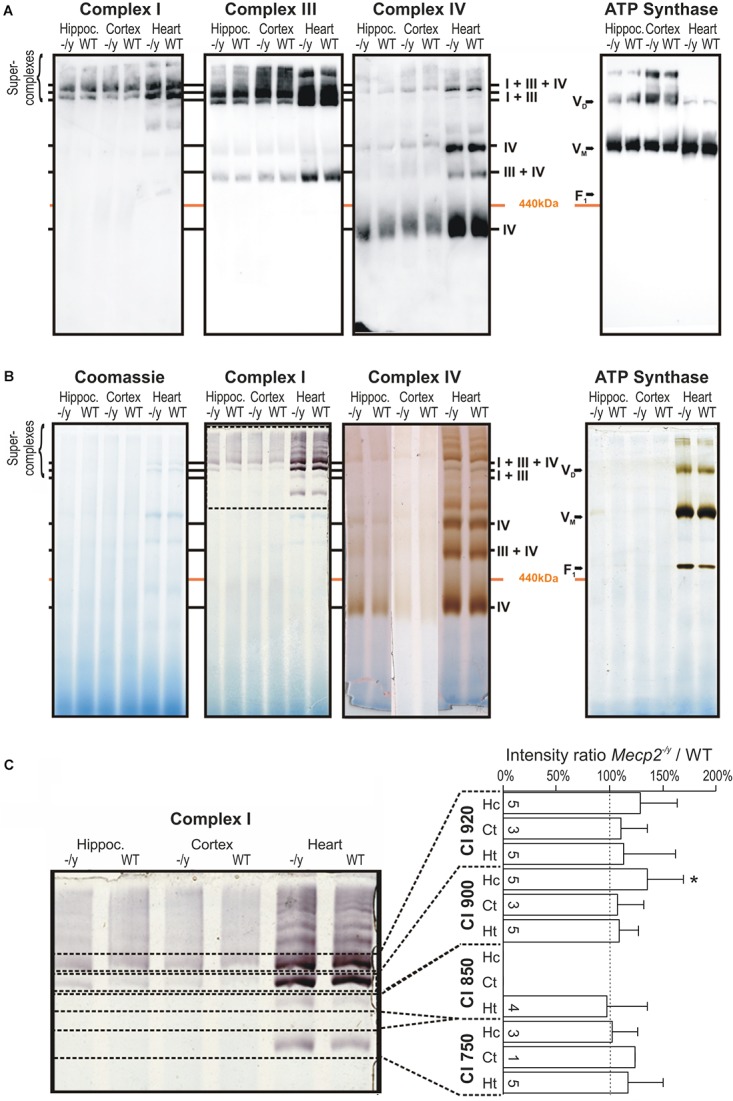
Pathological remodeling of the respiratory chain supercomplexes in mitochondria of *Mecp2^-/y^* mice is not evident. **(A)** Isolated mitochondria were solubilized in digitonin, separated by native gel electrophoresis (BN-PAGE), and respiratory chain complexes were analyzed by Western blotting. Due to limited space, *Mecp2^-/y^* tissue is denoted here as “*-/y”*. **(B)** Respiratory chain complexes were separated by BN-PAGE and stained for enzymatic activity of CI, CIV, and CV. Marked alterations for the respective respiratory complexes were not found. The section of the gel flanked by the dashed lines was analyzed in more detail in panel **C**. **(C)** Quantitative analysis of CI activity staining by densitometry does not reveal marked genotypic differences among WT and *Mecp2^-/y^* hippocampus. Dashed line indicates WT conditions, the number of samples (mice) analyzed is indicated (^∗^*p* > 0.05, one sample *t*-test).

Structural changes of the mitochondrial respiratory chain may provoke the generation of ROS (Chen et al., 2012; Vukotic et al., 2012). As roGFP1 imaging revealed exaggerated redox shifts of *Mecp2^-/y^* mitochondria in response to hypoxia and AMC, we screened for potential structural changes of the mitochondrial respiratory chain. We isolated mitochondria from hippocampus, cortex, and heart by differential centrifugation of tissue lysates (Argan et al., 1983; Dudek et al., 2016) and solubilized the isolated mitochondria in the mild detergent digitonin to preserve the supercomplex association. Solubilized protein complexes were then separated by native gel electrophoresis (BN-PAGE) and detected using antibodies against the core component of the complexes. CI was exclusively integrated in supercomplexes and the CI + CIII complex, which was visualized using antibodies against the CI subunit NDUFB8 in the high molecular range of the native gel. CIII and CIV were detected clearly in supercomplexes but were also present as smaller complexes (Figure 5A). ATPase was found in two complexes representing the monomeric V_M_ and the dimeric form V_D_. Obvious differences in the supercomplex-assembly among WT and *Mecp2^-/y^* tissues were not identified.

Therefore, we next analyzed the activity of the respiratory chain. The complexes were separated by BN-PAGE and subsequently their enzymatic activity visualized by in-gel activity staining. This method allows a direct correlation of their enzymatic activity with their position on the BN-PAGE gel. Specific staining for CI, CIV, and ATP synthase activity corresponded well with their positions in Western blot analyses, confirming specificity of the chosen antibodies (Figure 4, 5A). Activity staining showed most intense signals for heart, which also corresponds well to stronger signals in the Western blot approach (Figure 5B). Also for brain, prominent CI signals were obtained consistently. As CI showed a well distinguishable pattern, the individual bands (750, 850, 900, and 950 kDa) were specifically analyzed by densitometry and normalized to the respective WT pattern; pronounced differences were, however, not found (Figure 5C). Hence, despite detailed analyses of brain and heart tissues, the in-gel activities of CI, CIV, and CV did not markedly differ among WT and *Mecp2^-/y^* mice.

### Mitochondrial O_2_ Consumption Rates

**FIGURE 6:**
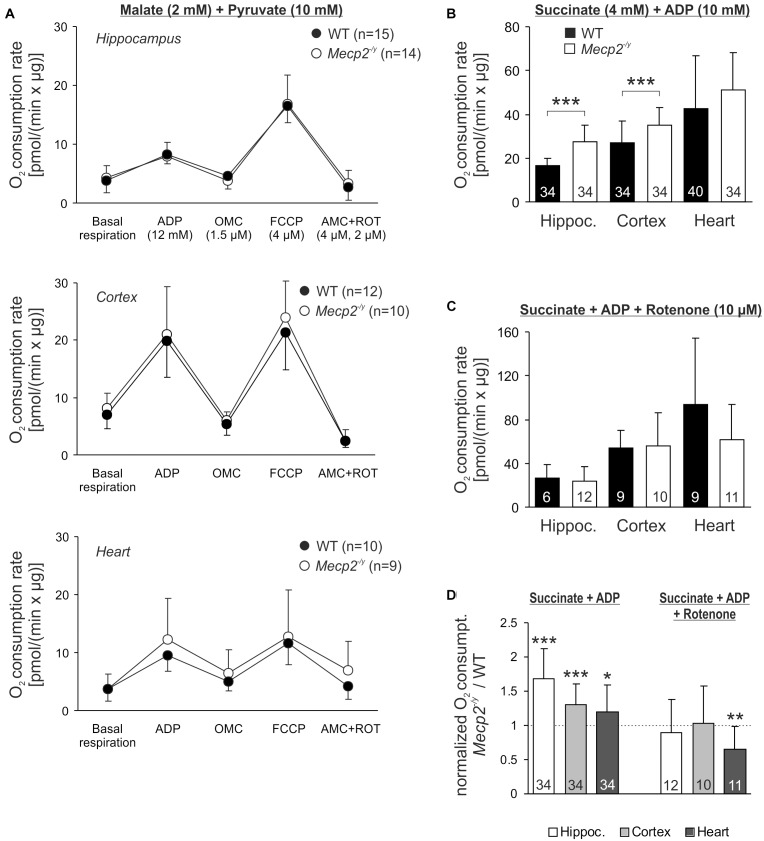
Intensified O_2_ consumption by mitochondria of *Mecp2^-/y^* brain in the presence of CII substrates. **(A)** O_2_ consumption rates of isolated mitochondria were measured at basal conditions in the presence of CI substrates. Subsequent measurements were performed after administration of ADP, the uncoupler FCCP and AMC plus rotenone. Neither basal respiration nor the other respiration states differed among WT and *Mecp2^-/y^* tissues. The number of samples (mice) analyzed is shown in the legend of each plot. **(B)** In the presence of ADP and the CII substrate succinate, O_2_ consumption was increased in *Mecp2^-/y^* hippocampal and cortical mitochondria as compared to WT (^∗∗∗^*p* < 0.001, unpaired two-tailed *t*-test). In heart, no changes were found. **(C)** Rotenone abolished the genotypic differences, suggesting that reverse electron flow from succinate into CI contributes to the increased O_2_ consumption of mitochondria from *Mecp2^-/y^* hippocampus and cortex. **(D)** The rotenone effect is especially clear, when the mitochondrial O_2_ consumption rates of *Mecp2^-/y^* samples are normalized to the respective WT conditions (^∗^*p* < 0.05, ^∗∗^*p* < 0.01, ^∗∗∗^*p* < 0.001; one sample *t*-tests).

Potential defects of the energy transmission in between complexes of the respiratory chain can be assessed only by quantifying the consumption of molecular oxygen. Isolated mitochondria from hippocampus, cortex and heart of WT, and *Mecp2^-/y^* mice therefore underwent real-time respirometry (Figure 6A). Mitochondrial suspensions were supplied with CI substrates (2 mM malate, 10 mM pyruvate) to define the O_2_ consumption rate of basal respiration. Adding ADP (12 mM) then markedly increased O_2_ consumption (state 3 respiration) to a similar extent in WT and *Mecp2^-/y^* samples (Figure 6A). Subsequent inhibition of ATP synthase (1.5 μM oligomycin) largely diminished O_2_ consumption in all samples, but did not reveal any genotype-related differences. FCCP application (4 μM) then evoked the maximal uncoupler-stimulated respiratory state, again, without revealing genotypic differences. Finally, mitochondrial respiration was blocked by AMC (4 μM) plus rotenone (2 μM), which almost completely abolished O_2_ consumption. The remaining fraction of residual O_2_ consumption did not differ either among *Mecp2^-/y^* and WT samples.

Recently, a dysfunction of CII was reported for female Rett mice (De Filippis et al., 2015). Therefore, we quantified mitochondrial O_2_ consumption also in the presence of the CII substrate succinate (4 mM). Indeed, respiration after addition of ADP (10 mM) was increased in *Mecp2^-/y^* hippocampus and cortex as compared to WT samples (Figure 6B). In heart, this genotypic difference was not evident. As reverse electron flux through complex I is one of the major mechanisms of mitochondrial ROS production (McLennan and Degli Esposti, 2000; Dröse, 2013), we included rotenone (10 μM) into the assay buffer. Under these conditions, which prevent a reverse electron transport from succinate, the increased O_2_ consumption rates in *Mecp2^-/y^* cortex and hippocampus were no longer evident (Figure 6C). This is in particular evident, when the normalized respiration rates (*Mecp2^-/y^* referred to WT) are plotted (Figure 6D).

### Mitochondrial ROS Production

**FIGURE 7:**
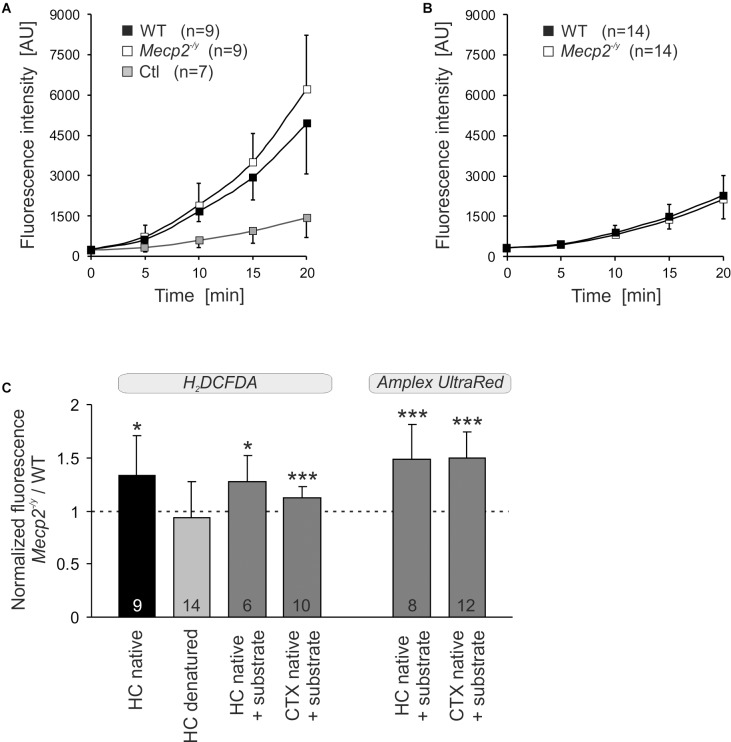
Increased ROS release from mitochondria of *Mecp2^-/y^* hippocampus and cortex. **(A)** ROS generation was quantified in mitochondrial suspensions under native conditions (assay buffer without substrates) using the oxidation-sensitive dye H_2_DCFDA. Genotypic comparison reveals an increased ROS formation by mitochondria from *Mecp2^-/y^* hippocampus. The control cuvettes (Ctl) contain buffer plus dye only and report the extent of photo- and autoxidation of H_2_DCFDA during the course of this assay. **(B)** In contrast, under denaturing conditions, neither a pronounced oxidation of H_2_DCFDA nor a genotypic difference among WT and *Mecp2^-/y^* hippocampus was observed. This confirms that it is indeed the native mitochondria, which mediate the marked oxidation of H_2_DCFDA in this cuvette assay. **(C)** Also with the substrates malate, pyruvate, and succinate included into the assay buffer, mitochondria from *Mecp2^-/y^* hippocampus (HC) and cortex (CTX) released higher amounts of ROS than WT mitochondria (dashed line). This was confirmed unequivocally also with the more reliable reagent Amplex UltraRed. For comparison of the different conditions tested, the normalized fluorescence ratios are plotted, by referring *Mecp2^-/y^* responses to the respective WT conditions. The number of samples (mice) analyzed is reported; asterisks mark statistically significant differences as compared to WT (one sample *t*-tests; ^∗^*p* < 0.05, ^∗∗∗^*p* < 0.001).

To assess whether the altered mitochondrial activities in *Mecp2^-/y^* hippocampus and cortex may give rise to an increased oxidative burden, we monitored the ROS generation of mitochondrial suspensions in a spectrofluorometric assay with the oxidation-sensitive dyes H_2_DCFDA and Amplex UltraRed. The extent and time course of dye oxidation depend on the amount of ROS generated, and were compared for isolated mitochondria from adult WT and *Mecp2^-/y^* brains. As both dyes may be prone to photo- and auto-oxidation, we also analyzed control cuvettes containing buffer plus dye but no mitochondria. Monitoring H_2_DCFDA fluorescence over 20 minutes showed a pronounced oxidation only in the native mitochondria-containing samples and a more intense ROS formation by *Mecp2^-/y^* mitochondria (Figure 7A). In contrast, in denaturing buffer (Triton X-100), we did not observe a marked oxidation of H_2_DCFDA nor any genotypic differences, confirming their dependence on native mitochondria with fully functional respiratory complexes as well as intact mitochondrial membranes (Figure 7B). For verification, we ran this assay also for native mitochondria in the presence of CI plus CII substrates (2 mM malate, 10 mM pyruvate, 4 mM succinate), and again observed a more intense ROS formation in *Mecp2^-/y^* than in WT hippocampal and cortical mitochondria (Figure 7C). For further confirmation, mitochondrial ROS formation was then rated also with the more reliable reagent Amplex UltraRed. This again verified a more intense ROS generation in native, energized mitochondria from *Mecp2^-/y^* hippocampus and cortex (Figure 7C).

### Body Temperature Measurements

Intensified mitochondrial metabolism may result in an increased body temperature, as part of the energy resting in the proton gradient may be converted into heat by regulated uncoupling (Nicholls and Locke, 1984; Ricquier and Bouillaud, 2000). Therefore, we measured the core body temperatures of WT and *Mecp2^-/y^* mice (∼PD50). On average, WT males had a body temperature of 37.4 ± 0.6°C (*n* = 11), whereas it was lower in *Mecp2^-/y^* males (35.7 ± 1.1°C, *n* = 12). Hence, a direct effect of the detected mitochondrial alterations on body temperature can be excluded, and the lower temperature of *Mecp2^-/y^* mice is likely a consequence of their markedly reduced body weights (WT 21.5 ± 1.2 g; *Mecp2^-/y^* 12.4 ± 2.1 g).

## Discussion

In the present study we quantified subcellular redox changes in hippocampal neurons, and assessed molecular changes of the individual respiratory complexes, their enzymatic activities, and their O_2_ consumption rates in brain and heart tissue. For these analyzes it was essential to ensure a complete lack of MeCP2 in each single cell being studied. Therefore, the heterozygous condition in female *Mecp2^+/-^* mice – which would have resulted in a mixed population of cells expressing and lacking MeCP2 – was circumvented by running all experiments on tissue isolated from male mice. Of course, RTT affects mostly females, but here disease mechanisms were studied on the (sub)cellular level. For a later *in vivo* assessment of redox-balance based therapies, it would of course be essential to include also female mice in the experiments, as only they truthfully represent the clinically relevant heterozygous condition.

### Redox Conditions in Mitochondrial Matrix and Cytosol

Subcellular redox conditions were imaged using the genetically encoded roGFP1 sensor (Dooley et al., 2004; Hanson et al., 2004). Whereas our previous studies still relied on lipofection (Großer et al., 2012; Bebensee et al., 2017), we now took advantage of our recently developed viral constructs (Can et al., 2017), to assure a highly neuron-specific expression of roGFP1. With this marked improvement, we specifically addressed redox balance in neurons of *Mecp2^-/y^* hippocampus, and obtained for the very first time a functional readout of redox conditions in living MeCP2-deficient cells at subcellular resolution. Clear compartmental differences became apparent. In WT neurons, mitochondria were more oxidized than the cytosol, which corresponds to smooth muscle (Waypa et al., 2010), and our findings in excitatory hippocampal neurons of adult redox-indicator mice (Wagener et al., 2016). Furthermore, in *Mecp2^-/y^* neurons, the cytosol was more oxidized than in WT, and it was even more oxidized than the mitochondrial matrix of *Mecp2^-/y^* neurons. In RTT, it therefore seems to be especially the cytosolic compartment, which is affected by an impaired and more oxidized redox balance.

In mitochondrial matrix, genotypic differences in redox resting baseline levels were not obvious. Nevertheless, mitochondria may challenge cytosolic redox balance by releasing oxidants. Superoxide is generated especially at CI and CIII (Boveris and Chance, 1973; Votyakova and Reynolds, 2001; Brand, 2010) and it accumulates not only in the matrix but also in the intermembrane space (Brand, 2010), from where it then may reach the cytosol. Indeed, earlier studies on isolated full brain and cerebellar mitochondria demonstrated by homovanillic acid/horseradish peroxidase-based spectrophotometric assays that mitochondria of female Rett mice generate larger amounts of H_2_O_2_ than those of WT mice (De Filippis et al., 2015; Valenti et al., 2017).

In line with these reports we observed a more intense oxidation of H_2_DCFDA and Amplex UltraRed by native *Mecp2^-/y^* hippocampal and cortical mitochondria. Despite being a common approach, H_2_DCFDA-assays need to be interpreted carefully, especially when applied in complex systems such as isolated tissues or intact cells. In contrast to Amplex UltraRed, H_2_DCFDA is not specific for H_2_O_2_ but responds also to hydroxyl radicals, peroxynitrite, and carbonate anion radicals. Furthermore, it may be oxidized by heme proteins or via Fenton chemistry by transition metals, and the enzymatic deacetylation of H_2_DCFDA as well as the oxidation of H_2_DCF may intrinsically generate ROS (Rota et al., 1999; Bonini et al., 2006; Wardman, 2007; Kalyanaraman et al., 2012). Therefore, we conducted the H_2_DCFDA assay in the simplified preparation of isolated mitochondria under native as well as denatured conditions and included respective controls.

In mitochondria-free buffers, an only slight oxidation of H_2_DCFDA occurred, ruling out transition metal contaminations of assay solutions or pronounced autoxidation. In denatured mitochondria (Triton-X100) a partial oxidation of H_2_DCFDA but no genotypic differences were seen. Therefore, some transition metal- or heme protein-mediated oxidation of H_2_DCFDA may have occurred in a genotype-independent manner. As most intense H_2_DCFDA oxidation and clear genotypic differences were evident in native mitochondria only, they can be assumed to depend largely on an intact inner mitochondrial membrane, a functional respiratory chain and mitochondrial membrane potential, and hence the ROS-formation inherent to mitochondrial (patho)physiology. Quantification or identification of the very oxidants involved is of course not possible with this assay for the above mentioned problems; also the obtained oxidation rates might be overestimated in view of the autocatalytic nature of the fluorescence dye used. For unequivocal confirmation, we therefore ran the Amplex UltradRed assay which specifically detects H_2_O_2_ (Zhou et al., 1997; Komary et al., 2010), and verified a more intense H_2_O_2_ release for native *Mecp2^-/y^* hippocampal and cortical mitochondria. In view of genotype-related differences, it therefore can be concluded that isolated native *Mecp2^-/y^* hippocampal and cortical mitochondria mediate a higher oxidative burden than WT mitochondria, which also includes the release of intensified amounts of ROS.

In addition to the differing steady-state redox baselines, acute redox challenges confirmed also a less stable redox homeostasis in *Mecp2^-/y^* cytosol and mitochondria. Blocking of CIII elicited more intense oxidizing responses in *Mecp2^-/y^* cytosol and mitochondria. Upon O_2_ withdrawal, both compartments showed more pronounced reducing shifts than those in WT neurons. In contrast, inhibiting SOD1 evoked less intense oxidations in *Mecp2^-/y^* cytosol and mitochondria than in WTs, which matches the dampened SOD1 activity found in RTT patient blood samples (Sierra et al., 2001). The cerebral redox-imbalance in RTT is, however, not limited to neurons. Just recently, we found the cytosol of *Mecp2^-/y^* hippocampal astrocytes to be more oxidized than in WTs (Bebensee et al., 2017).

The roGFP1 redox sensor contains exposed thiols, which adjust to ambient redox conditions. Therefore, it does not detect a particular oxidant, but rather reports general thiol redox balance, and it behaves as other cell-endogenous redox-sensitive proteins, which are controlled by redox-sensing thiols (Hanson et al., 2004; Meyer and Dick, 2010). Such redox regulation of protein function/activity was estimated to affect >800 (human) proteins (Weerapana et al., 2010). Accordingly, the redox imbalance in cytosol and mitochondria of *Mecp2^-/y^* hippocampus may tremendously disturb protein function via either too reducing or too oxidizing conditions. This would already suffice to provoke neuronal dysfunction and distort neuronal network properties, long before oxidative tissue damage occurs.

### Mitochondrial Mass and Protein Expression Levels

A crucial aspect, in which our mitochondrial assays differ from most earlier studies is that no sample pooling or full brain analyzes were performed. Instead, to detect subtle differences among *Mecp2^-/y^* and WT mice, we analyzed defined brain samples of individual mice. Also, we included heart tissue to assess mitochondrial function in another organ as well. Nevertheless, it is important to recall that even isolated from specified tissues of individual mice, mitochondrial suspensions derive from mixed cell populations, i.e., various neurons and glial cells in the case of brain samples.

Our Western blot analyses on hippocampus and cortex did not indicate any changes in mitochondrial mass. This is in line with MeCP2-deficient mouse fibroblasts and *MECP2* mutant human embryonic stem cells differentiated into neurons (Li et al., 2013; Shulyakova et al., 2017). Yet, we saw a trend toward increased mitochondrial mass in *Mecp2^-/y^* heart, and cell culture studies found higher mitochondrial contents in microglia (Jin et al., 2015) and hippocampal astrocytes of juvenile Rett mice (Bebensee et al., 2017). In part, an increased mitochondrial mass may serve to compensate the mitochondrial underperformance in RTT. However, this seems to be cell- and tissue-specific, and limited to certain developmental stages or experimental preparations. It certainly cannot explain in general the redox imbalance in RTT.

Rett mouse brains and patient-derived tissues present a heterogeneous spectrum of mitochondrial alterations (Müller and Can, 2014; Valenti et al., 2014; Shulyakova et al., 2017). Seemingly contradictory results arose especially from gene-arrays. An overall upregulation of the subunits of various respiratory complexes was found in blood (lymphomonocytes) of RTT patients (Pecorelli et al., 2013), whereas a genome-wide transcriptional repression, including various mitochondrial proteins, was evident in an embryonic stem cell model of RTT (Li et al., 2013). This emphasizes the importance of standardized procedures and the need of analyzing not only differential transcription, but also final protein levels. Fact is that despite potential candidate genes, details on actual changes in mitochondrial protein expression are still sparse. In late symptomatic male Rett mice, a downregulation of CI was seen in full brain mitochondria (Kriaucionis et al., 2006), and a lower expression of CIV was found in skeletal muscle (Gold et al., 2014). More recent whole brain studies on female MeCP2-308 mice did not reveal any obvious alterations of the individual mitochondrial complexes (De Filippis et al., 2015; Valenti et al., 2017). Instead, alterations were region specific, affecting the striatum in which CI and CIII showed lowered protein expression levels (De Filippis et al., 2015).

This regional specificity corroborates our results from symptomatic *Mecp2^-/y^* mice. CI (NDUFB8) was slightly less expressed in *Mecp2^-/y^* hippocampus and cortex than in WT, and CV (ATP5B) was slightly less expressed in *Mecp2^-/y^* cortex. Interesting in view of the CII-related intensified O_2_ consumption in *Mecp2^-/y^* hippocampus and cortex may be the trend toward an increased CII expression (SDHA) in these areas. Evidence for a lowered expression of CIV, as reported for *post mortem* frontal cortex of patients (Gibson et al., 2010), was not found in our mouse samples. Interestingly, in heart tissue no genotypic differences in the expression of the respiratory complexes were detected.

### Functionality and Stability of Respiratory Supercomplexes

The intensified mitochondrial ROS generation in Rett mice and the associated oxidative stress could aggravate mitochondrial dysfunction by destabilizing individual subunits of the supercomplex, abolishing facilitated electron channeling and releasing further ROS. However, steady state protein levels of the core components of the respiratory chain did not reveal noticeable changes. Neither were any obvious genotypic differences in the organization or enzymatic activity of mitochondrial supercomplexes detected in hippocampus, cortex and heart. Thus, a structural remodeling of the highly energy efficient supramolecular assemblies cannot be a cause for the limited mitochondrial performance in RTT.

This does not rule out, however, that the altered metabolic rate of isolated mitochondria may arise from a differential expression or altered enzymatic activities of some smaller regulatory subunits of these supercomplexes. Along this line, protein kinase A-dependent phosphorylation of CI (NDUFS4), which controls respiratory capacity and mitochondrial ROS formation, is decreased in the cerebellum of female Rett mice (De Filippis et al., 2015). Also elevated expression of the adenine nucleotide transporter 1 (ANT1) was proposed to cause an enhanced basal H^+^ leak of the inner mitochondrial membrane, followed by a compensatory increase of mitochondrial respiration (Shulyakova et al., 2017).

In-gel activity staining revealed no obvious genotype-specific defects in the enzymatic activities of CI, CIV, and CV. This suggests that at least in hippocampus, cortex and heart of *Mecp2^-/y^* mice, enzymatic activities of mitochondrial complexes are mostly intact. In line with this is also the unaltered ATP content we detected in the hippocampus of symptomatic *Mecp2^-/y^* mice (Fischer et al., 2009). Earlier studies on full brains mostly found reduced mitochondrial activities. Heterozygous female Rett mice (MeCP2-308) show lower CII and CV enzyme activities (De Filippis et al., 2015), whereas in *Mecp2^+/-^* mice CI, CII, and CV are less active (Valenti et al., 2017). In contrast, for *Mecp2^-/y^* mice a reduced CIV activity and an enhanced CIII activity were reported (Kriaucionis et al., 2006). Also skeletal muscle analyses revealed dampened enzyme activities of CII, CIII, and CIV in male MeCP2-deficient mice (Gold et al., 2014). Similarly, in patient muscle, succinate cytochrome *c* reductase (CII–CIII) and CIV activities are diminished (Coker and Melnyk, 1991). Analyzing cardiac muscle, we did not detect any changes in the enzyme activities of the respiratory complexes.

### Mitochondrial O_2_ Consumption Rates

Quantifying mitochondrial O_2_ consumption revealed no changes for *Mecp2^-/y^* heart, but increased rates for *Mecp2^-/y^* hippocampus and cortex fueled by CII substrates. This genotypic difference vanished in the presence of rotenone, which suggests that reverse electron flow into CI may be a contributing factor. As CII is not integrated structurally into the respiratory chain supercomplexes, we did not expect to observe any corresponding structural differences in the native gel electrophoresis. Others determined the O_2_ consumption rates of mitochondria from whole *Mecp2^-/y^* brain with polarographic O_2_ electrodes, and observed elevated rates for all respiration states (Kriaucionis et al., 2006). Also in *Mecp2^-/y^* microglia the O_2_ consumption is increased (Jin et al., 2015). In female Rett mice (MeCP2-308), increased respiratory rates were found in full brain-derived mitochondria fueled by complex II substrates (De Filippis et al., 2015; Valenti et al., 2017), indicating that mitochondrial defects also extend to the milder heterozygous genotype. In contrast, *MECP2* mutant human neurons, derived from stem cells, showed a lower basal O_2_ consumption, and reduced maximal respiration rate (Li et al., 2013), but these data were normalized to cell count and not to protein content.

As the observed reverse electron flow into CI may provoke maximal mitochondrial ROS production – especially with succinate as substrate (Votyakova and Reynolds, 2001) – this may well explain the more oxidized redox balance in *Mecp2^-/y^* hippocampal neurons, and similar conditions can be expected for cortical neurons. Others report intensified H_2_O_2_ formation by isolated brain mitochondria (De Filippis et al., 2015; Valenti et al., 2017) and elevated oxidative stress markers in blood, muscle and brain of Rett mice (De Felice et al., 2014; Gold et al., 2014).

A potential impact of the detected redox- and metabolic alterations on mitochondrial morphology remains to be elucidated. Our current focus was on functional aspects; nevertheless, this structural/functional interaction may be of interest as the RTT–related alterations in mitochondrial function may not necessarily be accompanied by gross alterations in their morphology (Kriaucionis et al., 2006).

## Conclusion

Our study confirms by quantitative subcellular redox imaging that the redox imbalance in RTT affects neurons and involves their cytosol and mitochondria. Major changes in the expression and enzyme activity of the respiratory complexes were not found, and a dissociation of mitochondrial supercomplexes was ruled out. Accordingly, massive mitochondrial defects and/or marked dysfunction of entire respiratory complexes can be excluded, which matches the observation that a pronounced neurodegeneration does not occur in RTT (Armstrong et al., 1995). Instead, only moderate alterations in mitochondrial function and/or mitochondrial regulation are to be expected, which may result in an inefficient electron flow within the respiratory chain, but do not threaten cellular viability. In line with this concept, mitochondria from *Mecp2^-/y^* hippocampus and cortex consumed more O_2_. This confirms the earlier proposed intensified mitochondrial respiration rates (Kriaucionis et al., 2006; Großer et al., 2012) and supports the concept that the mitochondrial underperformance in RTT may be considered an *“energy wasting state”* with increased O_2_ consumption but lowered ATP production (Jin et al., 2015). In view of these changes and the increased mitochondria mediated oxidative stress and ROS formation detected by us and others (De Filippis et al., 2015; Valenti et al., 2017), a causal chain appears more than likely. It spans from increased mitochondrial activity and O_2_ consumption, over exaggerated mitochondrial ROS release and cytosolic/mitochondrial redox imbalance to disturbed neuronal network function and a facilitation of disease progression.

Interestingly, alterations in mitochondrial morphology and metabolism with closely associated oxidative stress have also been confirmed for autism-spectrum disorders, Down syndrome and fragile X syndrome, which suggests that these alterations may be common to a broad array of genetic disorders with are closely associated with intellectual disabilities [see: (Rossignol and Frye, 2012; Valenti et al., 2014)]. Hence, a chain of common disease mechanisms may have to be considered, which emphasizes the importance of clarifying the detailed interrelation of mitochondrial alterations, redox imbalance and symptom severity.

In human neuroblastoma cells, a direct causal link of MeCP2 availability and mitochondrial function was proven by MeCP2 knockdown, which resulted in a diminished cytochrome *c* oxidase activity (Gibson et al., 2010). Accordingly, a therapeutic targeting of mitochondria appears promising. Indeed treating female Rett mice with the serotonin receptor 7 agonist LP-211 or the bacterial protein CNF1 (cytotoxic necrotizing factor 1) improved mitochondrial function and dampened their ROS production (De Filippis et al., 2015; Valenti et al., 2017). Therefore, it will be crucial now to define in detail the exact time course of mitochondrial impairment as well as the particular brain areas affected by these alterations and the downstream redox-imbalance. Only then, it will be possible to assess and judge reliably the potential merits of mitochondria- and redox-directed treatments in RTT.

## Ethics Statement

All experiments were in accordance with German regulations and authorized by the Office of Animal Welfare of the University Medical Center Göttingen as well as by the Lower Saxony State Office for Consumer Protection and Food Safety.

## Author Contributions

KC planned and conducted the redox-imaging experiments, analyzed the data and generated the figures, and contributed to writing of the manuscript. CM, LR, and GG planned and conducted the biochemical mitochondria-analyzes, analyzed the data and generated the figures, and contributed to writing of the manuscript. PR supervised all mitochondrial analyzes and commented data and manuscript. SK designed and generated the viral expression vectors and commented data and manuscript. JD co-supervised and planned the biochemical analyzes, commented data and manuscript, and contributed to manuscript writing. MM planned and supervised the entire project, finalized the data analyzes and data presentations, and prepared the final version of the manuscript. All authors read and agreed to the final version of the manuscript.

## Conflict of Interest Statement

The authors declare that the research was conducted in the absence of any commercial or financial relationships that could be construed as a potential conflict of interest.

## Abbreviations

ACSF: artificial cerebrospinal fluid
AMC: antimycin A
ATP5B: ATP synthase subunit beta
BN-PAGE: blue native polyacrylamide gel electrophoresis
CI complex I: NADH dehydrogenase
CII complex II: succinate dehydrogenase
CIII complex III: coenzyme Q-cytochrome *c* reductase
CIV complex IV: cytochrome *c* oxidase
CV complex IV: ATP synthase F_1_F_o_-ATPase
DEDTC: diethyldithiocarbamic acid
DIV: days *in vitro*
DTT: 1,4-dithio-DL-threitol
E_roGFP1_: roGFP1 reduction potential
FCCP: carbonyl cyanide 4-(trifluoromethoxy)phenylhydrazone
F_1_F_o_-ATPase: ATP synthase, complex V
GAPDH: glyceraldehyde-3-phosphate dehydrogenase
GSH: reduced glutathione
H_2_DCFDA: 2′,7′-dichlorodihydrofluorescein diacetate
HBSS: Hanks’-balanced salt solution
HEPES: 4-(2-hydroxyethyl)piperazine-1-ethanesulfonic acid
MAS: mitochondrial assay solution
MeCP2: methyl-CpG binding protein 2, protein
*MECP2*: methyl-CpG binding protein 2, encoding gene (human)
*Mecp2*: methyl-CpG binding protein 2, encoding gene (mouse)
*Mecp2^-/y^*: MeCP2-deficient male mouse (hemizygous)
*Mecp2^+/-^*: MeCP2-deficient female mouse (heterozygous)
NDUFB8: NADH dehydrogenase (ubiquinone) 1 beta subcomplex 8
NTB: nitrotetrazolium blue
OxD_roGFP1_: relative degree of roGFP1 oxidation
PD: postnatal day
PMSF: phenylmethyl-sulfonyl fluoride
roGFP1: reduction-oxidation sensitive green fluorescent protein 1
ROS: reactive oxygen species
R: fluorescence ratio (F_395_/F_470_)
R_ox_: ratio corresponding to full oxidation
R_red_: ratio corresponding to full reduction
RTT: Rett syndrome
SDHA: succinate dehydrogenase subunit A
SDS-PAGE: sodium dodecyl sulfate polyacrylamide gel electrophoresis
SOD1: Cu/Zn superoxide dismutase
TBS: Tris-buffered saline
WT: wildtype
VDAC3: voltage-dependent anion channel 3
